# Multiple Sources of Cholinergic Input to the Superior Olivary Complex

**DOI:** 10.3389/fncir.2021.715369

**Published:** 2021-07-15

**Authors:** Nichole L. Beebe, Chao Zhang, R. Michael Burger, Brett R. Schofield

**Affiliations:** ^1^Department of Anatomy and Neurobiology, Hearing Research Focus Group, Northeast Ohio Medical University, Rootstown, OH, United States; ^2^Brain Health Research Institute, Kent State University, Kent, OH, United States; ^3^Department of Biological Sciences, Lehigh University, Bethlehem, PA, United States

**Keywords:** acetylcholine, gerbil, pontomesencephalic tegmentum, modulation, arousal, plasticity, hearing, collateral

## Abstract

The superior olivary complex (SOC) is a major computation center in the brainstem auditory system. Despite previous reports of high expression levels of cholinergic receptors in the SOC, few studies have addressed the functional role of acetylcholine in the region. The source of the cholinergic innervation is unknown for all but one of the nuclei of the SOC, limiting our understanding of cholinergic modulation. The medial nucleus of the trapezoid body, a key inhibitory link in monaural and binaural circuits, receives cholinergic input from other SOC nuclei and also from the pontomesencephalic tegmentum. Here, we investigate whether these same regions are sources of cholinergic input to other SOC nuclei. We also investigate whether individual cholinergic cells can send collateral projections bilaterally (i.e., into both SOCs), as has been shown at other levels of the subcortical auditory system. We injected retrograde tract tracers into the SOC in gerbils, then identified retrogradely-labeled cells that were also immunolabeled for choline acetyltransferase, a marker for cholinergic cells. We found that both the SOC and the pontomesencephalic tegmentum (PMT) send cholinergic projections into the SOC, and these projections appear to innervate all major SOC nuclei. We also observed a small cholinergic projection into the SOC from the lateral paragigantocellular nucleus of the reticular formation. These various sources likely serve different functions; e.g., the PMT has been associated with things such as arousal and sensory gating whereas the SOC may provide feedback more closely tuned to specific auditory stimuli. Further, individual cholinergic neurons in each of these regions can send branching projections into both SOCs. Such projections present an opportunity for cholinergic modulation to be coordinated across the auditory brainstem.

## Introduction

The superior olivary complex (SOC) serves as a major computation center in the brainstem auditory system. It participates in a variety of brainstem auditory circuits and is a hub for many ascending and descending auditory pathways. Among the many functions SOC serves in hearing, its roles in sound localization are well known (Harrison and Feldman, 1970; Grothe et al., 2010). Ascending auditory inputs to SOC emerge from the cochlear nucleus (CN; Cant and Casseday, 1986; Kuwabara et al., 1991; Thompson and Schofield, 2000). In turn, ascending projections from the SOC project primarily to nuclei of the lateral lemniscus and the inferior colliculus (IC), with smaller projections to the superior colliculus and auditory thalamus (Schofield et al., 2014; Mellott et al., 2018; Mansour et al., 2021). The SOC neurons that are responsible for computing the location of sound sources in the azimuth plane include medial superior olive (MSO) and lateral superior olive (LSO) neurons (Helfert and Aschoff, 1996). To ensure computational stability and accuracy, these neurons establish a complex and precise neural circuitry (Adams and Mugnaini, 1990; Schofield and Cant, 1991; Smith et al., 1998). In this network, the role of excitation and inhibition in shaping sound-evoked responses are well studied using simple acoustic stimuli (Brugge and Geisler, 1978; Albrecht et al., 2014; Grothe and Pecka, 2014). However, in response to more complex stimuli, the ability to maintain computational stability and accuracy may be challenged. Elevated input intensity or complicated input components causes synaptic depression, and weakened synapses affect the timing and strength of signal transmission among these SOC neurons (Banks and Smith, 1992; Grothe and Sanes, 1993; Song et al., 2005; Kopp-Scheinpflug et al., 2008). In addition to the known excitatory and inhibitory inputs, neuromodulatory mechanisms may be available to modify the SOC network dynamically for optimized performance. Numerous studies have suggested that SOC neurons employ local neuromodulation to regulate synaptic transmission to accommodate the complexity of acoustic inputs. In the MNTB, a number of ion channels and/or receptors are involved in regulating the signal transmission at its large and highly reliable synapse from the calyx of Held (Kopp-Scheinpflug et al., 2011). In the MSO, GABA_B_ receptors modulate binaural synaptic inputs to ensure the precision of neural computation (Pecka et al., 2008; Hassfurth et al., 2010; Fischl et al., 2012). In the LSO, serotonergic modulation induces synaptic suppression of both excitatory and inhibitory inputs (Fitzgerald and Sanes, 1999).

The role of broad neuromodulatory systems that pervade most regions of the brain has received little attention at the level of the SOC. ACh regulates neural activity at several levels of auditory processing including the cochlea (Taranda et al., 2009; Ciuman, 2010), cochlear nucleus (e. g, Fujino and Oertel, 2001; Goyer et al., 2016; Kuenzel, 2019), inferior colliculus (Farley et al., 1983; Habbicht and Vater, 1996), thalamus (Sottile et al., 2017a,b) and cortex (Metherate, 2011; reviewed by Schofield and Hurley, 2018). Numerous reports suggest acetylcholine (ACh) is a neuromodulator of computational importance in the SOC. Receptor binding indicates that SOC nuclei have high levels of cholinergic receptors (Morley and Happe, 2000; Gahring et al., 2004; Happe and Morley, 2004). Developmental knock-out of alpha-7 nicotinic receptors suggests that ACh may contribute to temporal processing in the superior paraolivary nucleus (Felix et al., 2019). We have shown previously that, in the MNTB, ACh affects suprathreshold response magnitude, enhances near-threshold level discrimination, and enhances coding for signal in noise (Zhang et al., 2021).

Subcortical auditory centers derive their cholinergic input from two primary sources, the pontomesencephalic tegmentum (PMT) and the SOC. The PMT comprises the pedunculopontine tegmental nucleus (PPT) and the laterodorsal tegmental nucleus (LDT), both of which contain cholinergic and non-cholinergic neurons. The SOC contains multiple groups of cholinergic cells, including the well-known olivocochlear cells as well as lesser-known cholinergic cells that project to the cochlear nucleus or the MNTB (Sherriff and Henderson, 1994; Zhang et al., 2021). Cholinergic neurons in the PMT project broadly to CN, IC, and auditory thalamus (Schofield et al., 2011). We previously showed that the MNTB receives input from cholinergic cells of the SOC and the PMT (Zhang et al., 2021). In that report, we showed a tracer deposit restricted to the MNTB resulted in labeled cholinergic cells in the PMT and SOC. Larger tracer deposits that encroached on adjacent nuclei in the medial SOC produced similar results, but because none of those deposits excluded the MNTB, the sources of ACh input to other SOC nuclei were ambiguous. Here, we investigate cholinergic inputs to the SOC more broadly and we investigate the possibility of bilaterally branching projections from cholinergic cells of the SOC and the PMT into the SOC.

To identify the sources of cholinergic input to the SOC, we employed *in vivo* extracellular recordings to physiologically identify major nuclei in the SOC in the adult gerbil. Recordings were followed by injections of retrograde tracers (RetroBeads) to label innervating neurons. Among retrogradely labeled neurons, cholinergic cells were identified with an antibody to choline acetyltransferase (ChAT). Subsequent analysis revealed that both PMT and SOC provide cholinergic innervation of the ipsilateral and contralateral SOC. In addition, a small number of cholinergic cells in the lateral paragigantocellular nucleus (LPGi) project to at least some SOC nuclei. Individual cholinergic cells in each of these areas can send branching axons to innervate the SOC bilaterally. The results suggest a widespread cholinergic innervation of the SOC, with many SOC nuclei receiving cholinergic input from multiple sources, including cells that project bilaterally to the SOC.

## Materials and Methods

### Surgery and Perfusion

All procedures were conducted in compliance with Public Health Service and Institutional Animal Care and Use Committee (IACUC) guidelines. Adult Mongolian gerbils (*Meriones unguiculatus*) aged at least 3 months of either sex were used in all experiments. Tract tracer injections were made using methods described previously (Zhang et al., 2021). Initial anesthesia was administered with an intraperitoneal injection (5 ml/kg body weight) of a mixture consisting of 20% ketamine (100 mg/ml) and 2% xylazine (100 mg/ml) in 0.9% NaCl solution, yielding a final dose of 100 mg/kg body weight for ketamine and 10 mg/kg body weight for xylazine. The anesthetic depth was constantly monitored by assessing muscle tone and respiration rate. To maintain appropriate anesthesia, supplemental doses of anesthetic (0.05–0.10 ml) were injected subcutaneously every 30 min or whenever necessary. Subjects were transferred to a sound-attenuation booth (Industrial Acoustics) and mounted in a custom-made stereotaxic instrument. Body temperature was maintained at 37°C to 39°C by a heating pad through a homeothermic controller. One to three small craniotomies were performed on the interparietal bone caudal to the transverse sinus. The number of craniotomies depended on the number of injection targets. The dura was opened to expose the brain tissue.

Acoustic stimuli were digitally generated using TDT system III (Tucker-Davis Technologies) commanded through SPIKE, a custom-made software was used to collect spike times as well as analog chart recordings. The stimuli were attenuated (PA4/PA5; Tucker-Davis Technologies) and delivered to E.A.R. 3A earphones that are coupled to the external auditory meatus with tubes and calibrated using a $\frac{1}{4}$-inch free-field microphone and a microphone preamp (model 2221, Larson Davis). A low impedance glass search electrode (<1 MΩ) filled with 1M NaCl was first advanced using a remotely driven actuator into the brain stem to map the approximate borders of SOC nuclei. Major SOC subdivisions were identified based on differential noise-burst evoked responses. LSO neuron responses are evoked by ipsilateral sound stimulation and suppressed by contralateral sound (Boudreau and & Tsuchitani, 1968; Tollin and Yin, 2002). MSO neuron clusters are driven by either ipsilateral or contralateral sound (Goldberg and & Brown, 1969; Yin and Chan, 1990). Monaural MNTB neurons only respond to contralateral ear stimulation (Guinan and Li, 1990; Koka and Tollin, 2014). Because the MNTB is adjacent to VNTB and SPN, two other contralaterally driven SOC nuclei, the low-impedance search electrode was then replaced by a high impedance electrode (>5 MΩ) to record single-unit responses for a more precise identification of the MNTB population. Neurons with sustained sound-evoked responses that phase lock to low frequency stimulation were considered MNTB neurons. To ensure precise targeting, each population was demarcated from stereotaxic coordinates obtained from multiple search penetrations. Once the location was confirmed, the search electrode was withdrawn from the brain and replaced by a micropipette that was first backfilled with mineral oil and then front loaded with green or red RetroBeads (Lumafluor Inc.) diluted tenfold in 0.9% saline. Retrobeads were used because they are highly sensitive and are taken up minimally or not at all by fibers of passage unless the fibers are damaged (Katz et al., 1984; Schofield, 2008). To minimize damage to surrounding tracts, small deposits of diluted Retrobeads were made using a glass micropipette. Areas of tracer deposit showed minimal tissue damage under microscopic inspection, so we are confident that the vast majority of retrogradely-labeled cells had axon terminals in the tracer deposit site. For deposits directed at medial SOC, the electrode was lowered to the same coordinates identified for the MNTB whereas more lateral deposits were directed toward the coordinates identified for the LSO. Once the electrode was lowered to the desired depth, 100–200 nL of tracer was injected using a Nanoliter injector (World Precision Instruments). In a few cases, the same tracer was deposited *via* both medial and lateral locations to encompass a larger area of the SOC. In some animals, red beads were injected into the SOC on one side of the brain and green beads were injected on the opposite side. For this goal, separate micropipettes were used for each tracer to avoid cross-contamination.

After the retrograde tracers were deposited, the micropipette was removed and the craniotomy was covered with aseptic silicone gel and the incision was closed with Vetbond glue (3M). The animals then recovered on a heating pad under frequent monitoring for 24 h. Additional analgesic measures were applied during this period if necessary. After 48–72 h, the animals were injected with 0.2 ml/kg body weight Somnasol euthanasia solution (Henry Schein) intraperitoneally (yielding a final dose of 78 mg/kg body weight for pentobarbital sodium and 10 mg/kg body weight for phenytoin sodium) and perfused intracardially with 0.9% NaCl in 0.01 M phosphate buffer (PBS) followed by 4% paraformaldehyde in PBS. The brains were harvested and post-fixed in the latter solution at 4°C overnight. The brains were maintained in 30% sucrose PBS until processing for immunostaining.

### Immunohistochemistry

After removal of the cerebral cortex, the brain was frozen and cut on a sliding microtome into 40–50 μm sections in the transverse plane. Sections were treated in 0.4% Triton X-100 in PBS for 30 min (all steps at room temperature unless noted). After three 5-min washes in PBS, the tissue was treated with 20% normal rabbit serum (NRS) with 0.1% Triton X-100 in PBS for 1 h. Goat anti-ChAT polyclonal antibody (Chemicon AB 144P) was applied with 0.1% Triton X-100 and 1% NRS in PBS for 24–72 h at 4°C. The concentration of the primary antibody varied from 1:100–1:400. Following three 5-min washes in PBS, the tissue was incubated for 1 h with a secondary antibody (biotinylated rabbit anti-goat IgG, BA-5000, Vector Lab), at a 1:100 concentration with 1% NRS in PBS. Following three additional 5-min washes, tissue was incubated with an AlexaFluor 647-labeled streptavidin (1:100; Molecular Probes S- 21374) for 1 h at room temperature. The sections were rinsed in PBS then mounted on gelatin-coated slides and allowed to dry, then coverslipped with DPX (Aldrich Chemical Co., St. Louis, MO).

The anti-ChAT antibody recognizes choline acetyltransferase, the synthetic enzyme for acetylcholine found in cholinergic neurons. In guinea pig tissue, pre-adsorption with the ChAT peptide eliminated staining, and the antibody recognized a single band on a Western blot of guinea pig brainstem tissue (Motts et al., 2008). The pattern and appearance of ChAT staining reported here matched that seen in previous studies and in other species.

### Photography and Data Analysis

Photomicrographs of RetroBeads and ChAT-labeled cells were taken with a Zeiss AxioImager.Z2 microscope with an attached Apotome 2 to provide optical sectioning at 0.5 μm depth intervals. Low magnification images were taken using a 5× objective without the Apotome, while high magnification images were taken using a 63× oil-immersion objective (NA=1.4) with the Apotome. High magnification images shown are maximum intensity projections of image stacks. Adobe Photoshop was used to colorize and crop images and for global adjustment of levels. Plots of RetroBead- and ChAT-labeled cells were created with a Neurolucida system (MBF Biosciences) attached to a Zeiss AxioImager.Z1 microscope. Results from 14 tracer deposits in nine gerbils were used for analysis. We used anti-ChAT immunostaining to identify the Ch5 and Ch6 cholinergic groups, which mark the PPT and LDT, respectively (Mesulam et al., 1983). The LDT is located largely within the periaqueductal gray whereas the PPT extends through the pontomesencephalic tegmentum from a dorso-caudal location surrounding the superior cerebellar peduncle to a rostroventral location approaching the substantia nigra (Woolf and Butcher, 1986). The nuclei of the SOC are similar to those in guinea pigs and can be distinguished readily based on differences in background fluorescence of the cells and neuropil (Schofield and Cant, 1991). We are not aware of descriptions of the LPGi in gerbils, but we were able to identify the nucleus based on descriptions in other species (Andrezik et al., 1981; Kamiya et al., 1988; Stornetta et al., 2013). Every third section through the rostro-caudal extent of each area of interest was examined for ChAT+ cells, RetroBead-labeled cells, and cells labeled with multiple markers. The location of each labeled cell was plotted with a symbol indicating the labels present in the cell. After all the sections were plotted, the numbers of labeled cells were exported using Neurolucida Explorer, and were further analyzed in Microsoft Excel. Plots to show the distribution of labeled cells were exported from Neurolucida Explorer and figures were prepared with Adobe Illustrator CC.

## Results

### Injection Sites

**Figure 1:**
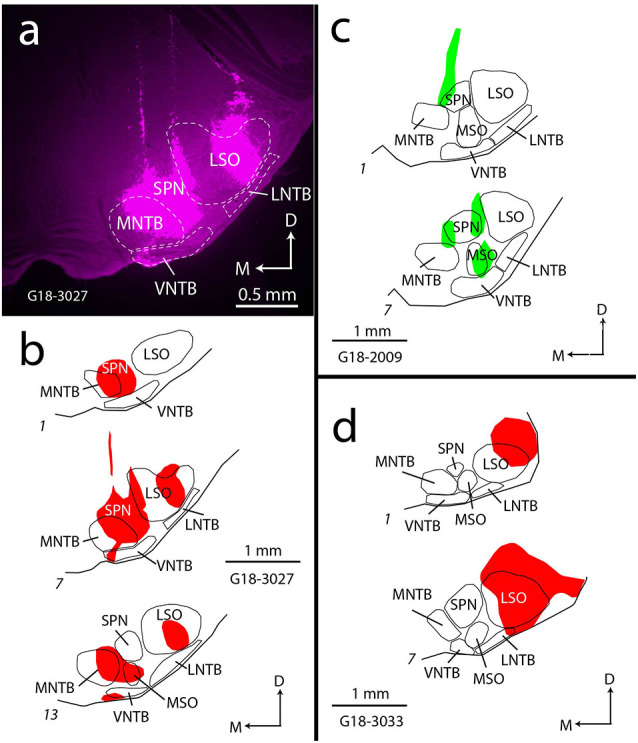
Examples of RetroBead injections in the SOC. **(A)** Photograph showing multiple deposits of red RetroBeads (magenta) in the SOC of case G18-3027. **(B)** Drawings depicting the rostro-caudal extent of the red RetroBead injection in case G18-3027. Section 7 corresponds to the photograph in panel **(A)**. This injection included most major SOC nuclei. In this and subsequent panels, sections are arranged from caudal (section 1) to rostral. **(C)** Drawings depicting the extent of the green RetroBead injection in case G18-2009. The deposits, in this case, were located in central parts of the SOC. **(D)** Drawings depicting the extent of the green RetroBead injection in case G18-3033. This deposit included only lateral parts of the SOC. Abbreviations: D, dorsal; LNTB, lateral nucleus of the trapezoid body; LSO, lateral superior olivary nucleus; M, medial; MNTB, medial nucleus of the trapezoid body; MSO, medial superior olivary nucleus; SOC, superior olivary complex; SPN, superior paraolivary nucleus; VNTB, ventral nucleus of the trapezoid body.

Each of the cases described here had deposits of RetroBeads that included various parts of the SOC. The large size of RetroBeads (on a molecular scale) often leads to irregular diffusion patterns and an irregular border of the deposit site (Schofield, 2008). In fact, a single injection can appear as multiple deposits in a single tissue section. As described in Methods, we frequently deposited RetroBeads at multiple sites in order to include a larger portion of the SOC, so it was essential to evaluate the entire SOC to identify the nuclei that were included in each experiment. Figure 1A shows an example of a large deposit of red RetroBeads. For this experiment, the beads were deposited *via* two penetrations at different medial-lateral locations. Figure 1B illustrates the spread of these deposits as seen in three different rostro-caudal levels through the SOC, showing that all major nuclei of the SOC were involved as were many periolivary nuclei. In other experiments, the deposits involved primarily medial SOC nuclei (Figure 1C) or lateral SOC nuclei (Figure 1D); the distributions are summarized in Table 1. In many cases, RetroBeads spread into the reticular formation just dorsal to the SOC, but the results in these cases did not differ from those with deposits restricted to the SOC.

**Table 1:** Summary of tracer deposits.

Case	MNTB	SPN	VNTB	MSO	LSO	LNTB
G18-2007 GB	XX	XX	XX			
G18-2007 RB	XX					
G18-2008 GB*	XX	XX	XX	XX		
G18-2009 GB*	X	XX		XX		
G18-2010 RB		XX				
G18-2010 GB	XX	XX				
G18-2011 RB	XX	XX		X		
G18-2011 GB	XX	XX				
G18-2012 RB*	XX	XX				
G18-2012 GB*	XXX	X				
G18-3027 RB*	XXX	XXX	XX	XX	XX	
G18-3030 RB*	XX	X		X	X	
G18-3030 GB	XX	X			XX	
G18-3033 RB*					XXX	X
G18-3033 GB*					XX	

For each tracer deposit, the extent of involvement of each SOC nucleus is indicated. Lack of markings indicates no involvement of a given nucleus, an “X” marking indicates minimal involvement of a nucleus, an “XX” marking indicates moderate involvement of a nucleus, and an “XXX” marking indicates extensive involvement of a nucleus. GB—green RetroBeads, LNTB—lateral nucleus of the trapezoid body, LSO—lateral superior olivary nucleus, MNTB—medial nucleus of the trapezoid body, MSO—medial superior olivary nucleus, RB—red RetroBeads, SPN—superior paraolivary nucleus, VNTB—ventral nucleus of the trapezoid body. *Indicates cases used for quantitative analysis.

Results were similar for red RetroBeads and green RetroBeads. Deposits of either tracer resulted in retrogradely-labeled cells in many auditory nuclei, including the cochlear nucleus and inferior colliculus, matching previous reports of inputs to the SOC (reviewed in Thompson and Schofield, 2000). Here, we focus on two questions regarding cholinergic inputs to the SOC. Our first goal was to identify the locations of cholinergic cells that project to the SOC, which we identified as cells that contained RetroBeads and were also immunopositive for choline acetyltransferase (ChAT), a selective marker of cholinergic cells. We focused on cholinergic cells in the PMT and the SOC, the primary sources of cholinergic input to the brainstem auditory nuclei (reviewed by Schofield and Hurley, 2018), and identified in our previous study of cholinergic inputs to the MNTB (Zhang et al., 2021). We also describe a small projection from the lateral paragigantocellular nucleus (LPGi) that projects to at least some of the SOC nuclei. Our second goal was to determine whether individual cholinergic cells send branching axonal projections (i.e., collateral projections) to both left and right SOC. For both goals, the presence of deposit sites within the SOC limited our ability to fully assess projections from within the injected SOC. Nonetheless, the results indicate that many of the SOC nuclei receive cholinergic inputs from multiple sources.

### The SOC Receives Cholinergic Input From Multiple Regions

Our previous report described cholinergic projections to the medial SOC, concentrating on the MNTB. Here, we expanded our study to include the entire SOC. In every case, the retrogradely labeled cells included both ChAT+ and ChAT-negative cells. In general, more cells were labeled after larger tracer deposits. While our goal was to assess inputs to the SOC overall, a few tracer deposits were limited to just one or two SOC nuclei; observations from these cases are described where relevant. Retrogradely-labeled cells in the PMT were quantified. Retrogradely-labeled cells are also described in the SOC and in the LPGi, however, cells in these areas were not quantified due to limited overall numbers of retrogradely-labeled/ChAT+ cells and because of proximity to the injection site.

#### Projections From the Pontomesencephalic Tegmentum

**Figure 2:**
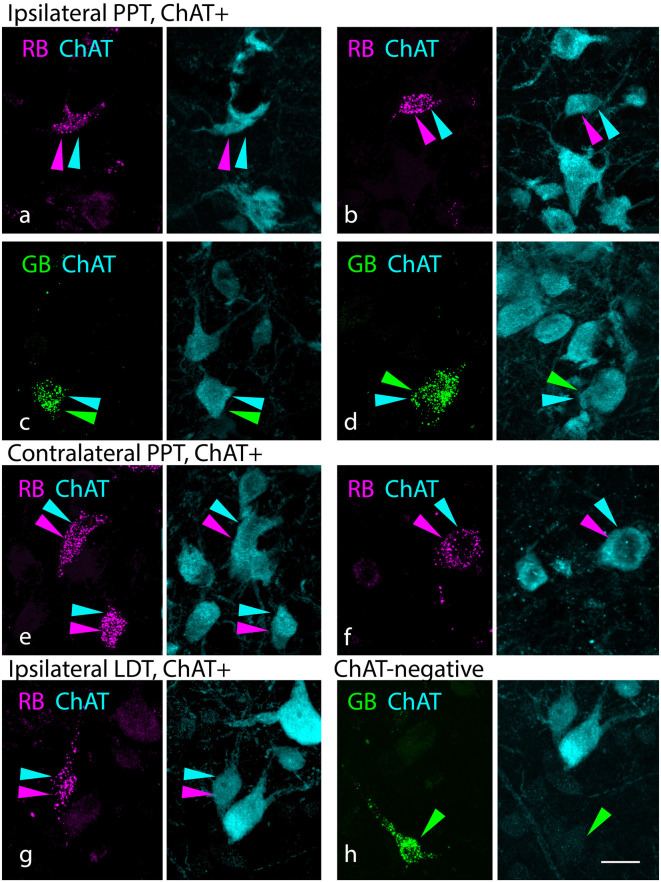
Cholinergic and non-cholinergic PMT cells project to the SOC. **(A–D)** Paired photographs show tracer-labeled cells (left panel, magenta or green) in the PPT ipsilateral to the injected SOC. The right panel in each pair shows the ChAT immunostain (cyan) from the same area, demonstrating that the tracer-labeled cells were also ChAT-immunopositive. Examples were seen with both red beads [“RB”, in **(A** and **B)**] and green beads [“GB” in **(C,D)**]. **(E,F)** ChAT+ cells labeled with RB in the PPT contralateral to the injected SOC. **(G)** Example of a ChAT+ RB-labeled cell in the ipsilateral LDT. **(H)** Example of a ChAT-negative, GB-labeled cell in the LDT contralateral to a GB injection. Panels **(A)** and **(F)** are from deposit G18-3033 RB (lateral SOC); the remaining panels are from deposit G18-2012 (medial SOC deposits). Scale bar = 20 μm. Abbreviations: LDT, laterodorsal tegmental nucleus; PMT, pontomesencephalic tegmentum.

Figure 2 shows cells in the PMT that were retrogradely labeled with red RetroBeads (“red beads”, RB) or green RetroBeads (“green beads”, GB). Many of these cells were ChAT immunopositive (ChAT+), suggesting they are cholinergic (Figures 2A–G). Other retrogradely labeled cells were clearly ChAT-immunonegative (“ChAT-negative”, Figure 2H). The presence of nearby cells with strong ChAT staining suggests that the lack of immunostaining in these retrograde cells was not due to failure of the immunostain (e.g., from lack of tissue penetration by the reagents). In addition to cholinergic cells, both the PPT and the LDT contain glutamatergic and GABAergic neurons, each of which could contribute to the ChAT-negative population (Wang and Morales, 2009; Boucetta et al., 2014; Kroeger et al., 2017). Similar results were produced by smaller tracer deposits, including deposits limited to the MNTB (G18-2007 RB; Zhang et al., 2021) as well as deposits restricted to the LSO/LNTB (G18 3033 RB) or the SPN (G18-2010 RB). Regardless of deposit size, ChAT-negative cells were among the tracer-labeled population, indicating that both cholinergic and non-cholinergic PMT cells project to the SOC nuclei.

For quantitative assessment of projections from the PMT nuclei, we chose eight deposits with the most substantial retrograde labeling along with robust immunostaining (Table 1). On average, 19–32% of retrogradely labeled cells were ChAT+ (Table 2). We conclude that both cholinergic and non-cholinergic cells in the PPT and LDT project to the SOC.

**Table 2:** Percentage of retrograde cells in each nucleus that were ChAT+.

Nucleus	Average	st dev	Maximum
Ipsilateral PPT	32%	20%	62%
Ipsilateral LDT	21%	17%	50%
Contralateral PPT	29%	18%	57%
Contralateral LDT	19%	13%	37%

*Summary of the percentage of retrogradely labeled PMT cells that were ChAT+. On average, 25–40% of retrogradely labeled cells were ChAT+. Data from eight tracer deposits (see asterisks in Table 1). The maximum column indicates the highest percentage of cells that were ChAT+ across all cases for each area. Total number of retrograde cells = 2,478. Total number of ChAT+/retrograde cells = 673*.

**Figure 3:**
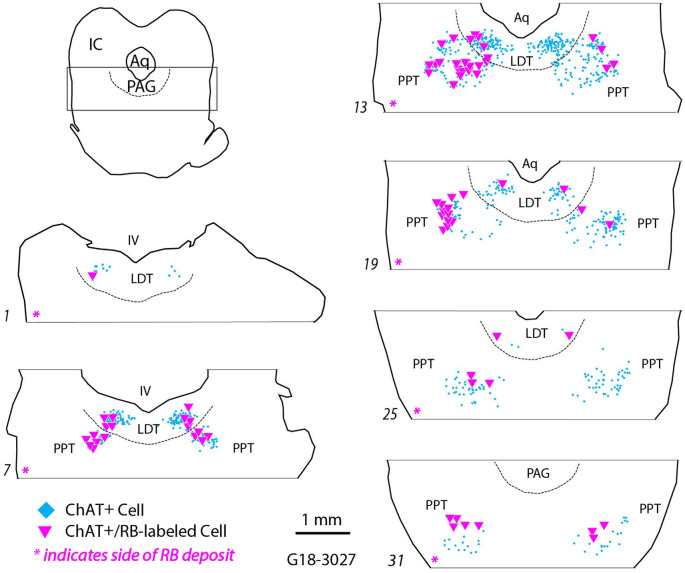
Retrogradely labeled cholinergic cells (magenta triangles) were located in the pontomesencephalic nuclei ipsilateral and contralateral to an injection of red RetroBeads in the left SOC. Each symbol represents a single labeled cell. ChAT+ cells that did not contain RetroBeads are illustrated (cyan diamonds) to indicate the extent of the pedunculopontine and laterodorsal tegmental nuclei (PPT and LDT, respectively). Numbered sections are arranged from caudal to rostral and represent the dorsal tegmental region (indicated by the rectangle in the orientation section). The dashed line indicates the ventral border of the periaqueductal gray (PAG). The magenta asterisk at the bottom of each section outline indicates the side ipsilateral to the RB deposit in the SOC. Aq, cerebral aqueduct; IC, inferior colliculus, IV, fourth ventricle.

Despite variation in tracer deposit size or involvement of different nuclei, the distribution of labeled cells in the PMT nuclei was qualitatively similar across cases. Figure 3 illustrates the distribution of ChAT+, RB-labeled cells (magenta triangles) in the PPT and LDT after a large injection (Case G18-3027; deposit site shown in Figures 1A,B). ChAT+ tracer-labeled cells were present bilaterally in PPT and LDT, with more cells ipsilateral than contralateral and, on each side, more cells in PPT than in LDT (Table 3).

**Table 3:** Distribution of tracer-labeled, ChAT+ cells in the PMT nuclei.

Nucleus	% of cells	st dev
Ipsilateral PPT	42%	13%
Ipsilateral LDT	17%	8%
Contralateral PPT	31%	18%
Contralateral LDT	10%	5%

*Summary of the distribution of ChAT+ retrogradely labeled cells in the four nuclei of the PMT. Data from eight tracer deposits (see asterisks in Table 1). Total number of retrograde cells = 2,478. Total number of ChAT+/retrograde cells = 673*.

#### Projections From the Superior Olivary Complex

**Figure 4:**
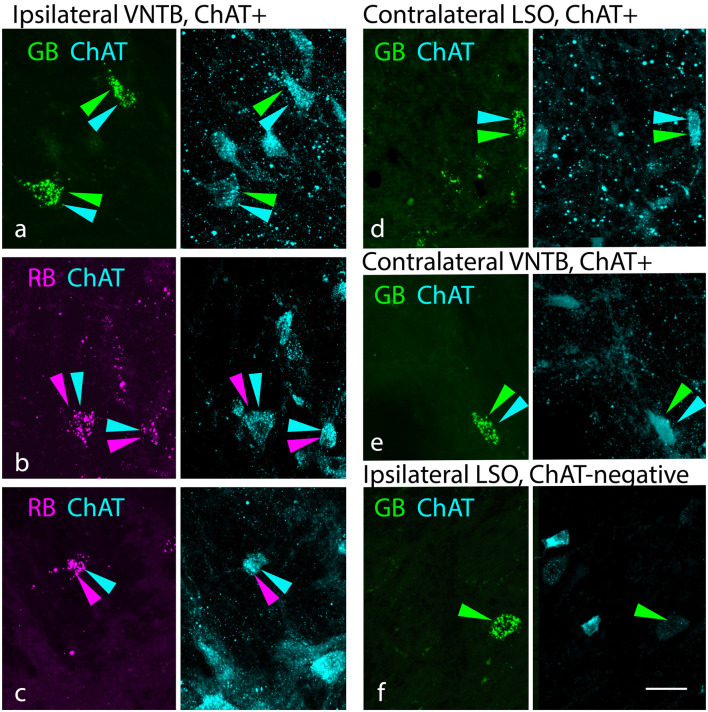
Cholinergic and non-cholinergic SOC cells were labeled by retrograde transport from the SOC. **(A–C)** Examples of ChAT+ tracer-labeled cells in the VNTB ipsilateral to the tracer deposit. Paired photographs show tracer-labeled cells (magenta or green, left panel) and ChAT immunostain (cyan, right panel), demonstrating that the tracer-labeled cells could be ChAT-immunopositive. Examples included cells labeled with green beads (GB) or red beads (RB). **(D,E)** Examples of ChAT+ tracer-labeled cells in the contralateral lateral superior olivary nucleus [LSO, panel **(D)** or contralateral VNTB (panel **E**)]. **(F)** Example of a GB-labeled ChAT-negative neuron in the LSO ipsilateral to a tracer deposit. LSO, lateral superior olivary nucleus; VNTB, ventral nucleus of the trapezoid body. Panels (**A,E,** and **F**) are from deposit G18-2012 GB (medial SOC deposit), panel **(B)** is from deposit G18-2011 RB (medial SOC deposit), and panels **(C)** and **(D)** are from G18-3033 (lateral SOC deposits). Scale bar = 20 μm.

**Figure 5:**
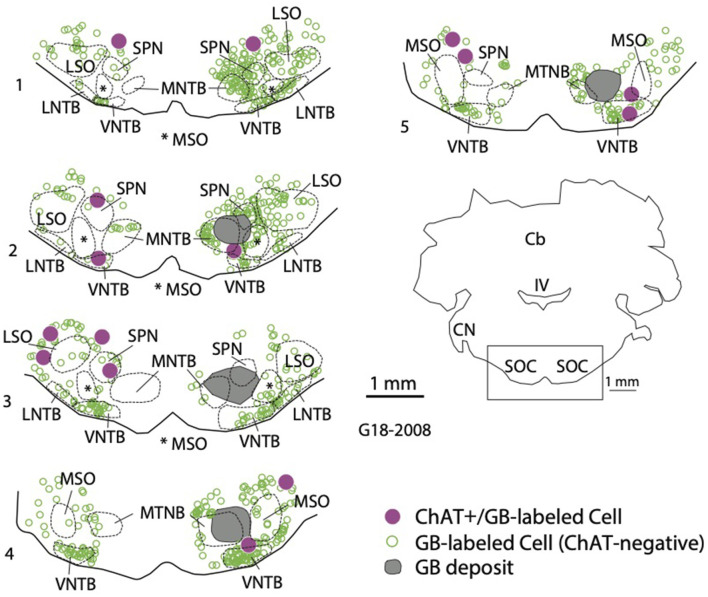
Retrogradely labeled cholinergic cells were located in the SOC both ipsilateral and contralateral to a tracer deposit. The plot shows a deposit of green beads (GB, deposit shown in gray) in the right SOC. GB-labeled cells that were ChAT+ (magenta circles) were scattered among SOC nuclei on both sides. In addition, a large number of GB-labeled cells that were ChAT-negative were also labeled (open green circles). Numbered sections are arranged from caudal to rostral and represent the ventral portion of each section to show the SOC (indicated by the rectangle in the orientation section). IV, fourth ventricle; Cb, cerebellum; CN, cochlear nucleus; LNTB, lateral nucleus of the trapezoid body; LSO, lateral superior olivary nucleus; MNTB, medial nucleus of the trapezoid body; MSO, medial superior olivary nucleus; SOC, superior olivary complex; SPN, superior paraolivary nucleus; VNTB, ventral nucleus of the trapezoid body.

Even though the tracer deposits obscured some of the SOC, it was possible to identify retrogradely labeled cells in parts of the SOC separated from the deposit sites. Such cells were numerous, reflecting well-known intra-olivary connections (reviewed by Thompson and Schofield, 2000). Figure 4 shows examples of tracer-labeled cells in the SOC ipsilateral or contralateral to a tracer deposit. Both ChAT+ (Figures 4A–E) and ChAT-negative (Figure 4F) tracer-labeled cells were observed. ChAT+ cells were scattered across the SOC, located among nearly all periolivary nuclei as well as within the LSO and around its borders (in the peri-LSO region). Similar results were observed after smaller tracer deposits. Deposits in the lateral SOC (LSO and LNTB) labeled ChAT+ cells in the ipsilateral and contralateral SOC (Figures 4C,D). A deposit restricted to the SPN also labeled ChAT+ cells bilaterally in the SOC. In all cases, tracer-labeled cells included ChAT-negative as well as ChAT+ cells. Figure 5 shows the distribution of ChAT+ and ChAT-negative retrograde cells (magenta and green, respectively) in the SOC after a deposit of green RetroBeads in the right SOC. Variation between cases was common; e.g., the VNTB often contained more ChAT+ retrograde cells than depicted in Figure 5. Such cells could also be clustered in an undefined region lateral to the MNTB, along the medial border of the MSO (a region noted to contain olivocochlear cells in gerbils; Aschoff et al., 1988). Across our cases, the only nucleus that never contained a ChAT+ retrograde cell was the lateral nucleus of the trapezoid body. It is likely that different cholinergic cells have different targets within the SOC, but further experiments will be needed to address this issue.

Small tracer deposits again provide additional information about cholinergic targets. Deposits in the lateral SOC (LSO and LNTB) labeled ChAT+ cells in the ipsilateral and contralateral SOC (Figures 4C,D). A deposit restricted to the SPN also labeled ChAT+ cells bilaterally in the SOC. In both cases, the labeled cells included ChAT-negative as well as ChAT+ cells. These cells were scattered among the olivary nuclei, similar to that seen after larger injections (i.e., in VNTB and LSO as well as other periolivary regions).

#### Projections From the Lateral Paragigantocellular Nucleus (LPGi)

**Figure 6:**
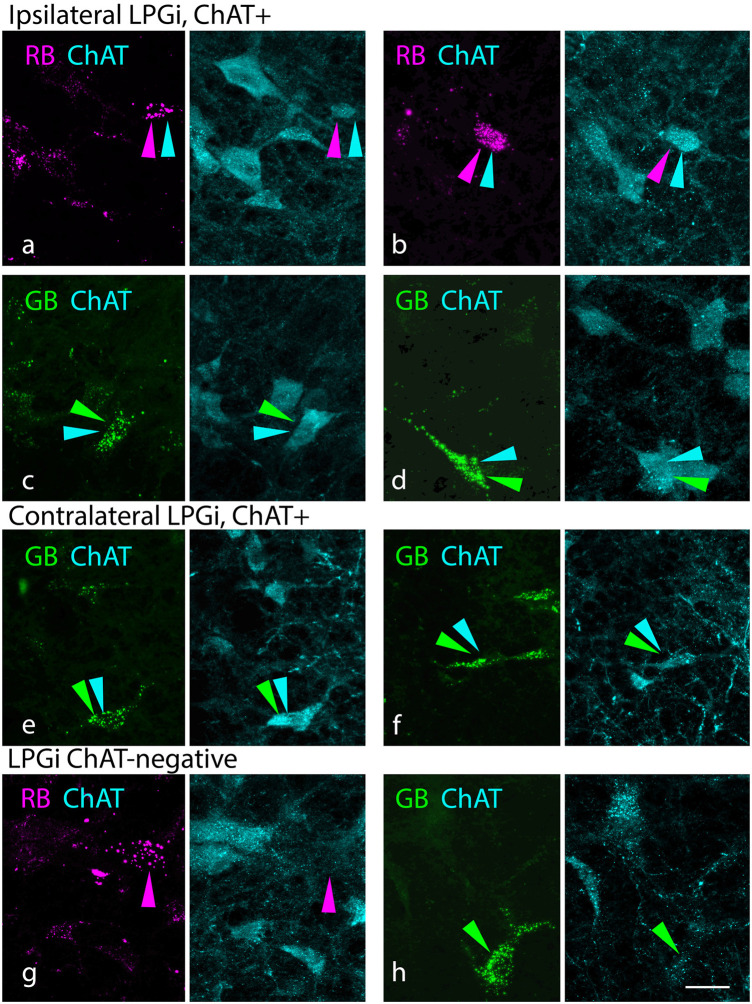
The lateral paragigantocellular nucleus (LPGi) contains cholinergic cells that project to the SOC. Paired photographs show tracer-labeled cells (magenta or green, left panel) and ChAT immunostain (cyan, right panel), demonstrating that the tracer-labeled cells could be ChAT-immunopositive. **(A–D)** ChAT+ cells labeled with red RetroBeads (RB; **A,B**) or green Retrobeads (GB, **C,D**) in the LPGi ipsilateral to a tracer deposit. **(E,F)** GB-labeled ChAT+ cells in the LPGi contralateral to the tracer deposit. **(G,H)** Tracer-labeled ChAT-negative cells in the LPGi. Panel **(B)** is from deposit G18-3033 RB (lateral SOC deposit); panel **(H)** is from deposit G18-3030 GB (medial SOC deposit); remaining panels are from deposit G18-2012 (medial SOC deposits). Scale bar = 20 μm.

As described above, our tracer deposits routinely labeled ChAT+ PMT and SOC cells. Given the spread of tracer deposits across cases, these results are consistent with cholinergic projections from these sources that terminate broadly throughout the SOC. Another area, the LPGi, has been reported in mice to project to several auditory brainstem areas, including parts of the SOC (Stornetta et al., 2013). The LPGi, a nucleus of the reticular formation also known as the medial rostral ventrolateral medulla, is located caudal to the SOC, just lateral to the medullary pyramid. This nucleus has been closely tied to autonomic and respiratory functions and has numerous connections with auditory structures (Andrezik et al., 1981; Kamiya et al., 1988; Bellintani-Guardia et al., 1996). In the present study, we found retrogradely labeled cells in the LPGi in some but not all cases. The LPGi is small (typically present in just one section in a one-in-six series). A single section generally contained just a few retrograde labeled cells. Like the PMT and SOC, the LPGi contains a variety of neurotransmitter phenotypes, and the retrogradely labeled cells included both ChAT+ and ChAT-negative examples (Figure 6). Such cells were observed ipsilateral and contralateral to the tracer deposit.

The presence of ChAT+ retrograde cells in LPGi did not appear to be related simply to the size of the tracer deposits. The small number of cholinergic cells in the LPGi may explain some of the variability, but another possibility is that the LPGi projections do not terminate throughout the SOC. The limited evidence available from mice suggests that LPGi projections to SOC terminate most densely in the LSO and along the dorsal margin of the SOC, with smaller projections to some of the other SOC nuclei (Stornetta et al., 2013). In the present study, we observed ChAT+ retrograde cells in the LPGi after large deposits restricted to medial or lateral SOC as well as a smaller deposit restricted to the SPN (case G18-2010 RB). The small injection in the MNTB (case G18-2007 RB) labeled cells in the LPGi, but none were ChAT+.

### Axonal Branching Allows Individual Cholinergic Cells to Innervate Left and Right SOC

**Figure 7:**
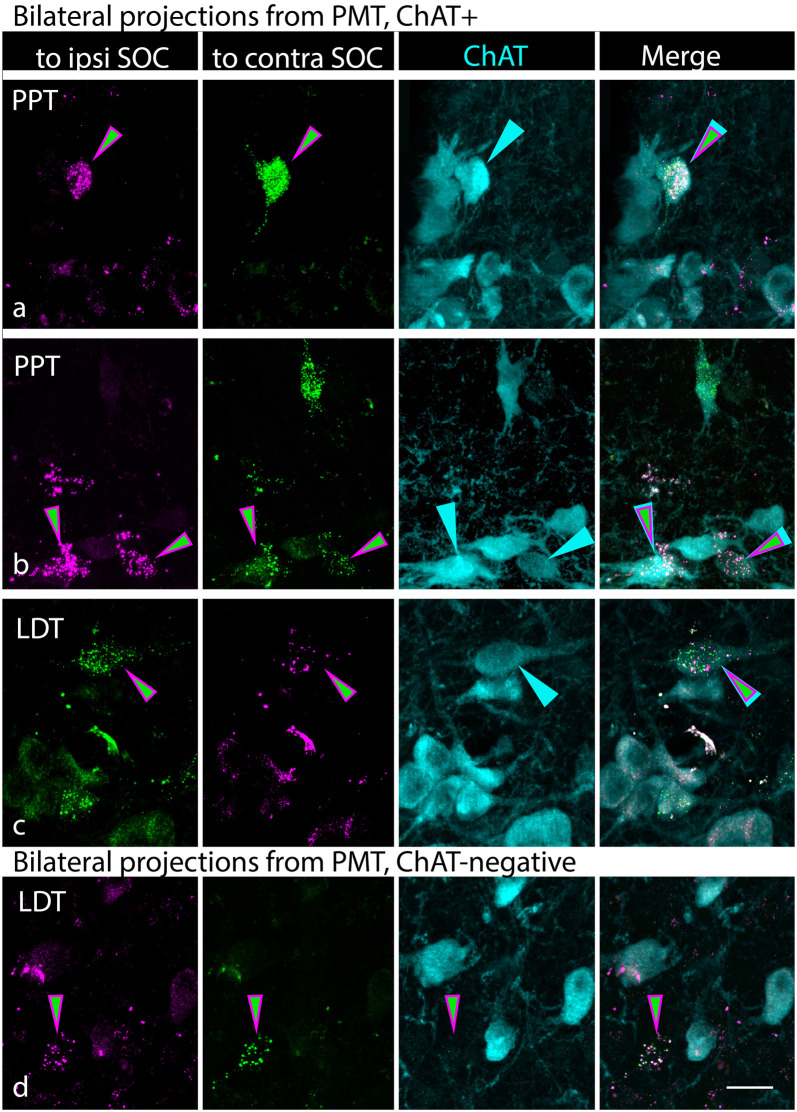
Cholinergic and non-cholinergic cells in the PMT send branching axonal projections to innervate the SOC bilaterally. **(A–C)** Each row of photographs shows a single field of view. The first two columns show the tracer label (RB in magenta; GB in green), with the first column showing the tracer injected into the ipsilateral SOC and the second column showing the tracer injected into the contralateral SOC (relative to the labeled cells). Magenta/green arrows identify cells that contain both retrograde tracers. Column 3 shows the ChAT staining, with arrows pointing to the same cells as in the first two columns. Column 4 shows the images merged, highlighting the triple-labeled cells. Examples are from the PPT **(A,B)** and LDT **(C)**. **(D)** A single cell in the LDT that contains both retrograde tracers, indicating a bilateral projection to the SOC, but is ChAT-negative (column 3), indicating it is unlikely to be cholinergic. All panels from case G18-2012. Scale bar = 20 μm.

Six of our experimental animals received bilateral injections, with RB in one SOC and GB in the opposite SOC. If an individual cholinergic neuron has an axon that branches to innervate both left and right SOC, we could expect to find such cells triple-labeled with the two tracers and the ChAT immunostain. We observed numerous cells in the PMT nuclei that contained both retrograde tracers; many, but not all, of these cells, were ChAT+. Figure 7 shows ChAT+, double-retrograde-labeled cells in the PPT (Figures 7A,B) and the LDT (Figure 7C). Triple-labeled cells were observed more often in the PPT than in the LDT, reflecting the pattern seen with single retrograde labeling. Figure 7D shows a double-retrograde cell in the LDT that was ChAT-negative. Such cells were observed in the PMT in all our cases with bilateral tracer deposits, suggesting that noncholinergic PMT cells also project bilaterally to the SOC.

**Figure 8:**
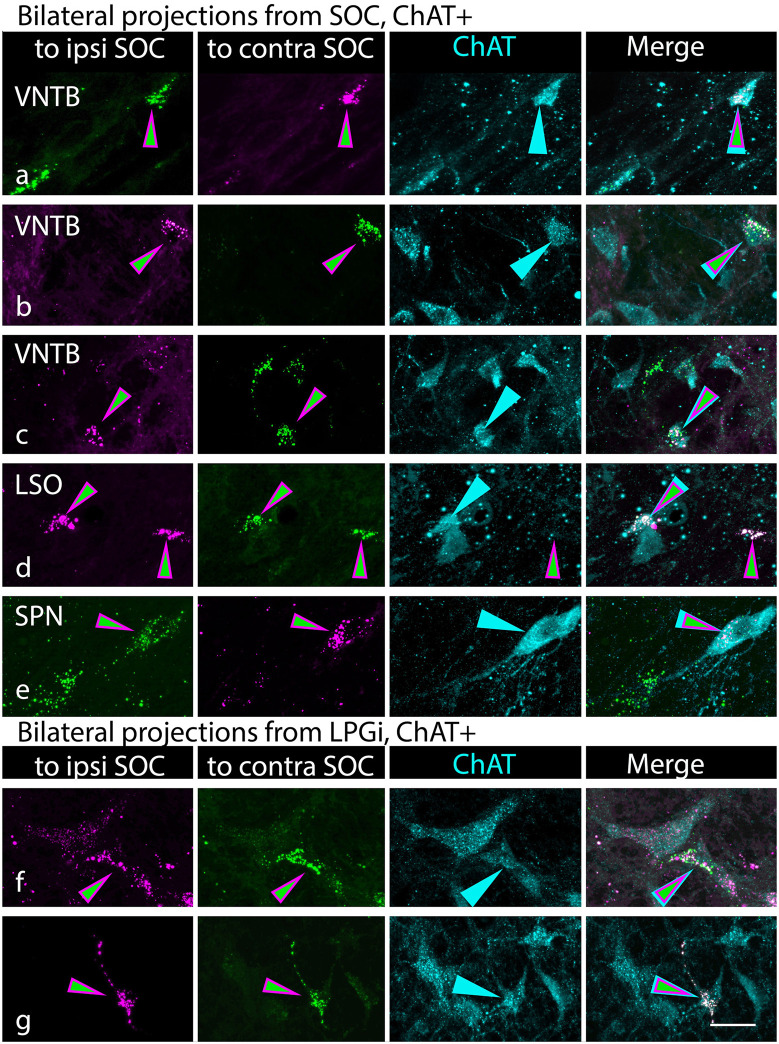
Cholinergic cells in the superior olivary complex (SOC) and lateral paragigantocellular nucleus (LPGi) send branching axonal projections to innervate the SOC bilaterally. Each row of photographs shows a single field of view. The first two columns show the tracer label (RB in magenta; GB in green), with the first column showing the tracer injected into the ipsilateral SOC and the second column showing the tracer injected into the contralateral SOC (relative to the labeled cells). Magenta/green arrows identify cells that contain both retrograde tracers. Column 3 shows the ChAT staining, with cyan arrows pointing to the same cells as in the first two columns. Column 4 shows the merged image, highlighting the triple-labeled cells. **(A–E)** Triple-labeled cells were found in numerous SOC nuclei, indicated by the labels in column 1. VNTB, ventral nucleus of the trapezoid body; LSO, lateral superior olivary nucleus, SPN, superior paraolivary nucleus. ChAT-negative cells could also be labeled with both retrograde tracers (panel **D**, cell on the right). **(F,G)** Triple-labeled cells in the LPGi. Panels **(A,B,** and **F)** are from G18-2012 (medial SOC deposits); panels **(C,D,** and **E)** are from G18-3033 (lateral SOC deposits); panel **(G)** is from case G18-3030 (medial SOC deposits). Scale bar = 20 μm.

Double-retrograde cells were also observed in the SOC. While the presence of the tracer deposits hindered full analysis of labeled SOC cells, there were clear examples of ChAT+, double-retrograde labeled cells in numerous SOC nuclei (Figures 8A–E). ChAT-negative double retrograde cells were also labeled (Figure 8D, cell on right); we focus here on the cholinergic (ChAT+) cells. Across cases, these cells were scattered across many of the SOC nuclei, including the LSO and various periolivary regions. Several of the examples shown in Figures 8C–E are from G18-3033, which had tracer deposits limited to the lateral SOC. In contrast, the examples in Figures 7A,B are from a case in which the tracer deposits focused on medial SOC nuclei (G18-2012; see Table 1). These results suggest that bilateral cholinergic projections can terminate in both the lateral and the medial SOC.

As described above, the LPGi contained retrogradely labeled, ChAT+ cells both ipsilateral and contralateral to the tracer deposit in several of our cases. Despite the small number of labeled cells in LPGi, we observed triple-labeled cells in cases with bilateral tracer deposits (Figures 8F,G), indicating that individual LPGi cholinergic cells can project bilaterally to the SOC. The number of cells was too small for quantitative analysis, but the fact that we observed triple labeled cells with a method that underestimates such projections (Schofield et al., 2007) suggests that bilateral projections may be a particularly common pattern for LPGi cholinergic cells.

## Discussion

**Figure 9:**
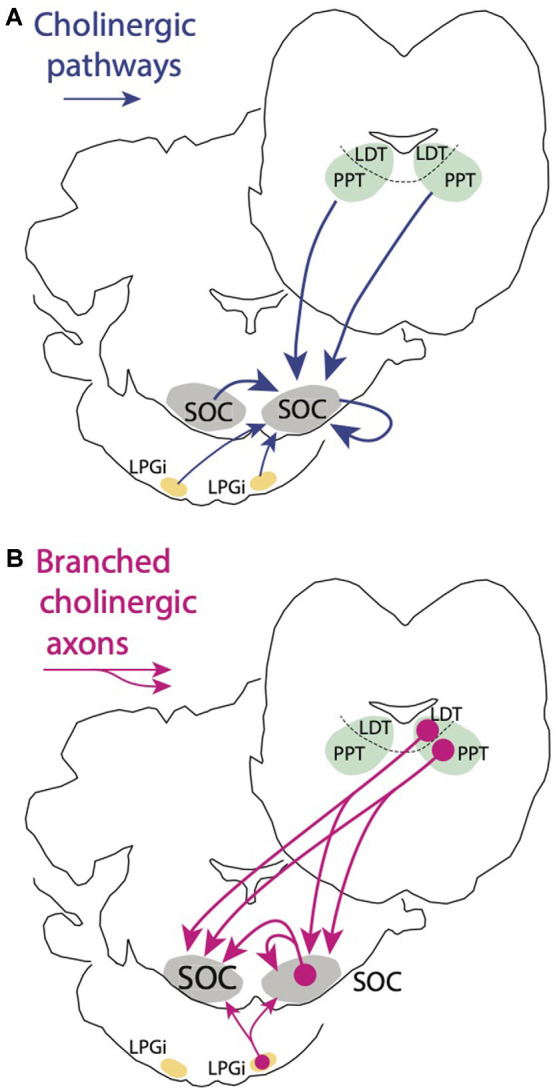
Schematic summarizing major findings from the present study. **(A)** Cholinergic inputs to each SOC originate from three areas. One area is the pontomesencephalic tegmentum (PMT), comprising the pedunculopontine tegmental nucleus (PPT) and the laterodorsal tegmental nucleus (LDT). Each SOC receives cholinergic input from both ipsilateral and contralateral PPT and LDT. The second source of cholinergic input to the SOC is from SOC cells themselves. Each SOC receives cholinergic input from neurons within the same SOC as well as from neurons in the contralateral SOC. A third, smaller source is the lateral paragigantocellular nucleus (LPGi). Both ipsilateral and contralateral LPGi project to the SOC. **(B)** A second finding is that individual cholinergic neurons can send branching axons to innervate the SOC bilaterally. Such collateral projections can arise from cholinergic cells in each of the regions that supply cholinergic innervation to the SOC (for simplicity, projections are shown only from one side).

Here, we’ve shown that the SOC receives cholinergic input from within the ipsilateral SOC, from the contralateral SOC, and bilaterally from the PMT (Figure 9). Projections from the PMT arise from both the PPT and the LDT, with ipsilateral projections more prominent and, on each side, PPT projections outnumbering LDT projections. Non-cholinergic PMT cells also appear to project to the SOC. Both cholinergic and non-cholinergic SOC cells also appear to project to many SOC nuclei. While non-cholinergic projections are especially numerous within the SOC, cholinergic SOC cells appear to innervate many of the SOC nuclei. Finally, a portion of the cholinergic cells that project to the SOC appear to have midline crossing axonal collaterals, allowing them to innervate both left and right SOC nuclei, presumably to provide a coordinated bilateral modulation of auditory processing in the SOC. In addition to the substantial cholinergic projections from the PMT and SOC, we document a smaller bilateral projection from the LPGi, a small nucleus of the reticular formation with connections to numerous auditory nuclei. Each of these regions, the PMT, the SOC, and the LPGi, provide cholinergic input to the SOC that likely serves a wide range of functions.

### Technical Considerations

The tracers and immunostains used here have been validated in previous studies and are unlikely to exhibit serious difficulties for interpretation (e.g., Motts and Schofield, 2010; Zhang et al., 2021). The main factor for consideration is the possibility of the retrograde tracer labeling axons of passage, i.e., axons that traverse but do not terminate in the area of the tracer deposit. As described in “Materials and Methods” section (Section “Surgery and Perfusion”), Retrobeads show little if any labeling of axons of passage unless those axons are damaged. We used fine glass micropipettes to limit such damage. Nonetheless, some axons may have been damaged during physiological recording or by the tracer micropipette. This concern was greatest for cells within the SOC that were injected, so we chose to report the presence and distribution of the labeled cells but not to quantify the labeled cells within the SOC. PMT cells are located a significant distance away from the SOC, so we had fewer concerns about quantifying the cells within these nuclei. The LPGi is located caudal to the SOC, so there is less chance that its axons were damaged unless they terminate in the SOC. Ultimately, confirmation of these findings will be from experiments based on chemically selective anterograde tracing of cholinergic axons from an identified source. Of the three sources of cholinergic input that we describe, the LPGi is the only one so far confirmed by anterograde transport methods (Stornetta et al., 2013). Similar confirmation will be needed for the cholinergic projections to the SOC from cells in the PMT and in the SOC itself. Such experiments will also provide important information about the density and distribution of cholinergic projections from each of these sources.

### Effects of ACh in the SOC

Our previous work suggested multiple roles for ACh in the MNTB, including effects on suprathreshold response magnitude, enhancement of near-threshold level discrimination, and enhanced coding of signal in the noise, with evidence that the MNTB receives cholinergic inputs from both the SOC and the PMT (Zhang et al., 2021). In the nearby SPN, mice lacking the cholinergic α7 nicotinic receptor show delayed sound-evoked responses and degraded spike precision (Felix et al., 2019). Aside from these studies, there is little direct information about the effects of ACh in SOC nuclei. In the cochlear nucleus (CN), which sends auditory information into the SOC, the roles of ACh have been studied more extensively. The effects ACh has in the CN vary by both CN region and cell type. For example, in the dorsal CN (DCN), ACh can alter neuronal sensitivity, affect spontaneous firing rates, and affect synaptic plasticity (Chen et al., 1998; Zhang and Kaltenbach, 2000; Zhao and Tzounopoulos, 2011). Blocking muscarinic signaling in the fusiform cell layer of the DCN affects spontaneous activity and alters stimulus timing-dependent plasticity; given the ties between these processes and evidence of tinnitus, cholinergic signaling in the DCN may be altered in tinnitus (Stefanescu and Shore, 2017). In T-stellate cells of the ventral CN (VCN), ACh contributes to sound-evoked excitation and may play a role in the encoding of spectral peaks in noise (Fujino and Oertel, 2001; Oertel et al., 2011). In spherical bushy cells, a different VCN cell type, cholinergic signaling plays a role in setting resting membrane potential, increases dynamic range and increases temporal precision (Goyer et al., 2016). The roles of ACh in the SOC likely vary based on the nucleus and cell type.

The effects of ACh are dependent on the subtypes of ACh receptors expressed by SOC cells. Many authors have described moderate or high expression of the nicotinic α7 subunit in the SOC (Morley et al., 1977; Hunt and Schmidt, 1978; Clarke et al., 1985). As described above, mice lacking the α7 subunit have delayed evoked responses and decreased spike timing precision in several auditory nuclei, including the SPN (Felix et al., 2019). The β4 nicotinic subunit is also highly expressed in the SOC (Gahring et al., 2004), and the relatively rare α5 nicotinic subunit is highly enriched in the SOC, especially in the SPN, compared to other brain regions (Wada et al., 1989). This all points to a variety of nicotinic acetylcholine receptors (nAChRs) being especially dense in the SOC. Muscarinic acetylcholine receptors (mAChRs) are also present in the SOC (Glendenning and Baker, 1988). There is less information about which subtypes of mAChRs are present, although both M2 and M3 receptors seem to be expressed in SOC nuclei (Safieddine et al., 1996; Yao and Godfrey, 1997). Activation of nAChRs (cation channels) typically elicits fast depolarization. Calcium permeability, ligand affinity, and channel kinetics can all differ based on subunit makeup for nAChRs (Gharpure et al., 2020). In contrast, mAChRs are G-protein coupled receptors and their activation typically elicits slower responses. Activation of mAChRs can lead to depolarization or hyperpolarization depending on the associated G proteins. mAChRs have been shown to regulate synaptic plasticity and circuit activity throughout the brain (Fernández de Sevilla et al., 2020). Despite their “slow” kinetics (compared to nAChRs), mAChRs may also contribute significantly to temporal processing, even in temporally demanding auditory cell types (Kuenzel, 2019). Based on receptor expression profiles, we would expect both nicotinic and muscarinic effects throughout the SOC.

### Functional Diversity of Sources of Cholinergic Projections to the SOC

A key finding in the present study is the identification of multiple sources of cholinergic input to the SOC: the PMT, cholinergic cells in the SOC itself, and for at least some SOC nuclei, inputs from cholinergic cells in the LPGi. It is likely that projections from each of these regions serve different functions.

#### Cholinergic Sources From Within the SOC

The present study identified cholinergic cells scattered throughout the SOC that innervate SOC nuclei on both sides of the brain. These cholinergic SOC cells clearly overlap in distribution with olivocochlear cells, but to the best of our knowledge, there is no evidence for olivocochlear cells to send axonal branches to any SOC nucleus. Medial olivocochlear cells (MOCs) situated in medial parts of the SOC send projections into the cochlea that synapse on outer hair cells to affect the cochlear amplifier (Schofield and Beebe, 2020). MOCs also send branches into the CN (Benson and Brown, 1990; Brown et al., 1991), and may be the source of cholinergic inputs onto T-stellate cell dendrites. As discussed above, cholinergic inputs to T-stellate cells have been shown to enhance the encoding of signal in noise (Fujino and Oertel, 2001), much like cholinergic inputs to the MNTB (Zhang et al., 2021). MOC branches in the CN may also serve to convey information about cochlear gain (Brown et al., 1988). If cholinergic innervation of the SOC comes in part from MOC branches, we would expect it to serve similar purposes, either enhancing signal in noise or conveying information about outer hair cell activation. Given the role of MOCs in modulating the cochlear amplifier, an intra-olivary projection might serve to modulate the SOC neuron threshold or gain to compensate for alterations in input from the ear.

Lateral olivocochlear cells (LOCs) are situated in lateral parts of the SOC and synapse in the cochlea on the afferent terminals of spiral ganglion neurons, where they meet inner hair cells (Schofield and Beebe, 2020). LOCs are more heterogeneous and less well-understood than MOCs, however, there is some evidence that they might send axon branches into the CN, and specifically to different regions of the CN than MOCs (Ryan et al., 1990). Even at the level of the cochlea where they have been most studied, the functions of LOCs are not well-understood (Frank and Goodrich, 2018).

The SOC also contains a group of small cholinergic cells in the VNTB that project to the CN and lack the characteristic morphology of MOCs (Sherriff and Henderson, 1994). Targets of these cells appear to include cells of the acoustic nerve nucleus, suggesting a role in early (and rapid) responses to startling stimuli (Gómez-Nieto et al., 2008). Here, we demonstrate that the SOC receives cholinergic input from within the ipsilateral SOC and from the contralateral SOC, however, it is unclear which cholinergic groups (MOCs, LOCs, or non-olivocochlear) participate in these projections. Based on the wide mediolateral distribution of cholinergic SOC cells that make SOC projections, we hypothesize that multiple cholinergic groups in the SOC may be involved.

#### Cholinergic Cells in the PMT

Cholinergic cells of the PMT are implicated in a variety of processes. As part of the ascending reticular activating system, they are active during waking and REM sleep and less active during slow-wave sleep (Boucetta and Jones, 2009). PMT cells also function in motor control, sensory gating, reward association, and attention (Garcia-Rill, 1991; Winn, 2006; Yeomans et al., 2006). Activation of cholinergic PMT cells enhances the startle response, in keeping with its wider roles in arousal (Azzopardi et al., 2018). PMT projections to auditory nuclei might serve to generally increase auditory responses during periods of increased arousal or may have a more selective response, perhaps enhancing neuronal responses only to certain salient sounds.

Cholinergic PMT cells project to many auditory nuclei, including the medial geniculate body, the inferior colliculus, and the CN (Steriade et al., 1988; Motts and Schofield, 2010; Mellott et al., 2011). Many PMT cells respond to auditory stimuli, but in contrast to SOC neurons, the PMT neurons tend to be broadly tuned for frequency and often adapt quickly to a repeated stimulus (Reese et al., 1995a,b). Some PMT neurons show a longer latency response to auditory stimuli, perhaps related to descending inputs from the auditory cortex (Reese et al., 1995a,b; Schofield and Motts, 2009). Projections from the auditory cortex have been implicated in cortically-driven plasticity of subcortical auditory neurons, including neurons in the CN, IC, and MG (reviewed in Schofield and Beebe, 2019). The present results raise the question of cortically-driven cholinergic effects in the SOC.

#### Cholinergic Cells in the Lateral Paragigantocellular Nucleus (LPGi)

The LPGi is well connected to other auditory nuclei: it receives input from the CN, IC, and the auditory cortex, and it projects to the CN and the IC (Andrezik et al., 1981; Kandler and Herbert, 1991; Van Bockstaele et al., 1993; Bellintani-Guardia et al., 1996). However, none of these studies marked the cholinergic cells in the LPGi, so it is unclear to what extent they are involved in the auditory circuits. The LPGi contains other neurotransmitter phenotypes (e.g., serotonin, GABA) and some of these cells may correspond to the ChAT-negative LPGi cells labeled by the tracer in the present study, but again it is impossible to relate these projections to the physiology of the cells. Complicating speculation is the unclear relationship of the auditory components in LPGi vs. autonomic components (e.g., Carrive and Gorissen, 2008; Koganezawa et al., 2008; Dergacheva et al., 2010). Auditory functions may be focused in the rostral LPGi and autonomic functions more caudally, but this remains to be confirmed physiologically (Andrezik et al., 1981). Further insight into the functions of the cholinergic LPGi cells will require more data on the response properties of these cells and their specific targets in the SOC and other auditory nuclei.

### Functions of Bilateral Innervation *via* Branching Cholinergic Axons

At its simplest level, a branching axon allows an individual neuron to influence two (or more) distant targets. Widespread axonal branching can allow for a relatively small population of neurons to exert effects across a large portion of a pathway, supporting global adjustments of neuronal sensitivity, for example, in response to an arousing stimulus. Such branching is common among modulatory systems, including cholinergic projections from the PMT (Descarries and Mechawar, 2008). Within the subcortical auditory system, cholinergic PMT cells can send collateral projections to targets on the two sides of the brain (e.g., left and right IC), to targets at different hierarchical levels of the auditory pathway (e.g., to IC and auditory thalamus), or to a combination (bilateral and multilevel; Schofield et al., 2011). Bilateral projections from individual PMT cholinergic cells to left and right SOC demonstrated in the present study, provide another example of widespread cholinergic projections. It will be interesting in future studies to determine whether these cholinergic cells also innervate other auditory structures, extending the span of PMT cholinergic projections from the SOC to, perhaps, the thalamus.

We also observed bilateral projections from SOC and LPGi cholinergic neurons. For further insight into their function, more information is needed about the cells giving rise to these projections. Under what conditions are these cells active? How broad are their projections within the SOC? Do they project to additional targets outside the SOC? While collateral branching could provide an opportunity for widespread effects, restricted projections may indicate highly specific effects on the target cells.

### Conclusions

A key issue from the discussions above is that cholinergic projections from different sources are likely to serve different functions. Projections from the PMT are likely to be activated in association with arousal and top-down modulation, perhaps contributing to plasticity driven by higher functions. Projections from the SOC are more likely to be narrowly tuned for auditory stimulus parameters and to serve as a feedback function for the earliest stages of auditory processing, from the cochlea to CN and SOC. Some cholinergic functions may be similar across SOC nuclei (e.g., the need to adjust neuronal sensitivity in response to reduced afferent input), but other functions may be more narrow, associated with individual nuclei and especially with individual cell types. The plethora of ACh receptor types could allow for varied functions within and across nuclei. A key step for future studies will be to identify the receptor types associated with specific cell types and with specific auditory circuits. Another important step will be to trace cholinergic pathways into the SOC with anterograde tracing methods. The retrograde tracing experiments here suggest widespread projections from each cholinergic area. Visualizing the terminations of each pathway will provide valuable information about the nuclei and cell types targeted by each source of cholinergic input.

## Data Availability Statement

The original contributions presented in the study are included in the article, further inquiries can be directed to the corresponding author.

## Ethics Statement

The animal study was reviewed and approved by IACUC Lehigh University.

## Author Contributions

NB, CZ, RB, and BS performed the research. NB, CZ, RB, and BS wrote the manuscript. All authors contributed to the article and approved the submitted version.

## Conflict of Interest

The authors declare that the research was conducted in the absence of any commercial or financial relationships that could be construed as a potential conflict of interest.
